# The Role of Occupational Health Services in Psychosocial Risk Management and the Promotion of Mental Health and Well-Being at Work

**DOI:** 10.3390/ijerph18073632

**Published:** 2021-03-31

**Authors:** Aditya Jain, Juliet Hassard, Stavroula Leka, Cristina Di Tecco, Sergio Iavicoli

**Affiliations:** 1 Nottingham University Business School, University of Nottingham, Nottingham NG8 1BB, UK; aditya.jain@nottingham.ac.uk; 2 School of Medicine, University of Nottingham, Nottingham NG8 1BB, UK; Juliet.Hassard@nottingham.ac.uk; 3 Cork University Business School, University College Cork, T12 K8AF Cork, Ireland; 4 Department of Occupational and Environmental Medicine, Epidemiology and Hygiene, Italian Workers’ Compensation Authority INAIL), 00078 Rome, Italy; c.ditecco@inail.it (C.D.T.); s.iavicoli@inail.it (S.I.)

**Keywords:** occupational health services, psychosocial risk management, mental health, well-being, health system, occupational safety and health

## Abstract

The development and enhancement of occupational health services (OHS) at the national level is central to ensuring the sustainable health, well-being and work engagement of the working population. However, due to differences in national health, social security and occupational safety and health systems, the content, capacity, coverage and provisions of OHS vary considerably across national contexts. Obtaining a better understanding in terms of such similarities and variations internationally is essential as such comparative information can help inform evidenced-based decision-making on OHS at both policy and practice levels. This paper therefore reviews and analyses the key policies, standards and approaches in OH systems and services, using both academic and grey literature, across 12 industrialised countries (Australia, Canada, Finland, France, Germany, Ireland, Italy, Japan, The Netherlands, Poland, United Kingdom and the United States of America). It provides a detailed overview and categorization of OHS in these selected countries in terms of the legal and policy context, organisation and financing and coverage and staffing while specifically discussing variations aimed at psychosocial risk management and the promotion of mental health and well-being at work. It draws conclusions on key development needs of OHS internationally to ensure psychosocial risk management and mental health promotion are prioritised effectively in a preventive manner.

## 1. Introduction

Demographic changes, recent economic downturns and the impact of the Covid-19 pandemic, paired with rapid technological changes and a rapidly ageing working population, are defining features of the contemporary labour market [1,2]. Such immediate and forthcoming challenges are anticipated to result in a shortage of skilled labour, reduction in work ability and adverse impact on the health, safety and well-being of the workforce [2,3]. Consequently, the importance of sustainable employment is a growing area of interest to policy makers [4] and organisations alike [5,6]. Several studies over the past decades have produced comprehensive scientific evidence which demonstrates the impact of adverse employment (precarious employment and unemployment) and working conditions (chronic exposure to occupational hazards and adverse psychosocial work environments) on health [7]. 

The psychosocial work environment relates to psychosocial factors at work which are aspects of work organisation, design and management that include, among others, work demands, the availability of organisational support, rewards and interpersonal relationships in the workplace. These factors do not immediately carry a negative or positive connotation. However, when reference is made to psychosocial hazards then it is implied that these aspects of work organisation, design and management have the potential to cause harm to individual health and safety (e.g., poor mental health, burnout, heart disease, musculoskeletal disorders) as well as other adverse organisational outcomes such as sickness absence, reduced productivity or human error [3,8]. Whether an organisation will have a positive or negative psychosocial work environment will depend on how effectively it manages psychosocial risk-the potential of psychosocial hazards to cause harm [9]. In a wider perspective, psychosocial risks are a major public health concern and are associated with economic and social security challenges [10]. Preventing and managing psychosocial risks are important now, but will become even more important in the post Covid-19 world, where workers will be increasingly exposed to psychosocial hazards arising from the present and future uncertainty of the work situation or from changes in work processes and arrangements, and their negative consequences on workers’ well-being and mental health [1,11]. 

One way in which health and work ability of the working population can be addressed is by strengthening the provision of occupational health services (OHS) [12]. The development and enhancement of OHS at the national level is central to ensuring the sustainable health, well-being and work engagement of the working population [13]. This approach has the aim of proactively managing risks for health and safety before serious outcomes affect the workforce (e.g., depression, anxiety), prioritising prevention and reducing the burden of disease. However, there is limited research which examines the role played by OHS in psychosocial risk management and the promotion of mental health and well-being at work. This paper therefore aims to do so by examining the context, systems, structure, coverage and capacity of OHS while specifically discussing variations aimed at psychosocial risk management and the promotion of mental health and well-being at work in a selection of industrialised countries across the world.

### Occupational Health Services and Their Role

The International Labour Organization (ILO, Geneva, Switzerland) Occupational Health Services Convention (No. 161) defines OHS as, “services entrusted with essentially preventive functions and responsible for advising the employer, the workers and their representatives in the undertaking on the requirements for establishing and maintaining a safe and healthy working environment which will facilitate optimal physical and mental health in relation to work and the adaptation of work to the capabilities of workers in the light of their state of physical and mental health”. The focus of OHS systems is therefore on three different objectives:

(i) the maintenance and promotion of workers’ health and work capacity;
(ii) the improvement of the working environment and work to become conducive to safety and health;
(iii) development of work organisations and working cultures in a direction which supports health and safety at work and, in doing so, also promotes a positive social climate and smooth operation, and may enhance the productivity of the enterprises [13].

Convention No. 161 also considers OHS as an integrated, comprehensive, multidisciplinary team containing all the elements needed for the improvement of health at work, improvement of the working environment, promotion of workers’ health and the overall development of the structural and managerial aspects of the workplace needed for health, safety and well-being [14]. The provision of OHS thereby means carrying out activities in the workplace with the aim of prevention, protection and promotion of workers’ safety, health and well-being, as well as improving working conditions and the working environment. These services are provided by occupational health professionals functioning individually or as part of special service units of the enterprise or of external services [15]. OHS must therefore be multidisciplinary and multi-sectoral, involving, in addition to occupational health and safety professionals, other specialists both within the enterprise and outside, as well as competent authorities, the employers, workers and their representatives [16]. 

OHS systems can play a unique role in the sustainability of the welfare state, as they bridge health policies and other social policies (education, environment, mobility, social protection, etc.), which influence the determinants of health of people. Occupational health as part of national policy emphasises the importance of work on social protection and funding arrangements of OHS and statutory accident insurance. Improvements in working conditions and the working environment make contributions to national development and make up part of successful economic and social policies [17]. In addition, OHS and employers make up an important part of prevention, protection and promotion of health as part of health policy and public health. In most industrialised countries across the world, the main responsibility for prevention measures and promoting occupational health and safety at enterprise level lies with the employer. Employers may cover the costs of OHS and make a necessary contribution to cover the costs of ill health and to improve the health, safety and well-being at work in enterprises [18]. 

National health systems and policies supervise and support employers in their duties. These systems often include a tripartite approach based on social dialogue between workers and employers, enforcement of legal provisions by the competent safety and health authorities (through labour inspection), support provided through occupational health and prevention services, including services provided by social security institutions, etc. [19,20]. Consequently, because of these differences, the content, capacity and coverage of OHS systems vary considerably across national contexts. 

In accordance with national conditions and practice, OHS may be organised by individual enterprises or groups of enterprises concerned (in-plant, group or industry specific model), public authorities or official services (hospitals/clinics, primary healthcare), social security institutions (social security model) or any combination of these models. As a rule, legislation allows flexibility in the choice of structural models of OHS in order to meet local conditions and practices, which can be either mandated directly by law, be voluntary and market driven or a combination of both. In some countries, governmental control and economic incentives are used to direct the extent and content of services [13], but in most the funding of OHS is typically arranged by employers and driven by the market. This is particularly true in countries where OHS provision is voluntary in nature. The provision of OHS by employers might be viewed as a financial burden, however, economic cost-benefit analyses have shown that investment in OHS typically yields a return on investment by improved productivity and profitability [21].

Recent research highlights that while many countries across the world have drawn up policies, strategies and programmes for OHS, there are gaps in implementation, capacity and coverage of such provisions. The infrastructures and institutional and human resources for the implementation of strategies remain insufficient in the majority of countries (implementation gap). While the estimated coverage of services is low, with only a quarter of the total global employed population having access to OHS (coverage gap). The content and multidisciplinary nature of OHS corresponds to international guidance, but the coverage, comprehensiveness and content of services remain largely incomplete due to a lack of infrastructure and shortage of multi-professional human resources (capacity gap) [22]. Obtaining a comprehensive understanding of the similarities and variations in the context, systems, structure, coverage and capacity of OHS will highlight some of the key development needs of OHS internationally to ensure psychosocial risk management and mental health promotion are prioritised effectively in a preventive manner, and help in addressing some of these gaps.

## 2. Methodology

Within this context, this study carried out an analysis of international case studies of OHS systems using a systematic, iterative step-wise approach. Framework analysis, typically used in policy-level research, was the analytical approach used to inform the employed research strategy [23]. This process involved three key stages: preparation, data collection and data analysis. The developed methodological framework sought to systematically identify, extract, appraise, synthesise and comparatively evaluate key sources of information and evidence. 

### 2.1. Preparation

The preparation stage involved a narrative-style review, examining both the academic and grey literatures. As part of this process, the national contexts, welfare models and health care systems were examined and categorised based on the taxonomy adapted from Hämäläinen [18]. The result of the literature review directly informed the selection of a diverse set of national cases to examine, and a research protocol to guide the development and analysis of case studies. The research protocol outlined a search strategy, clarified the structure and conceptual focus to case studies, and provided a data extraction template to standardise data analysis procedures and describe the analysis framework. 

The selection of national contexts was carried out to ensure inclusion of countries across the range of welfare state regimes and diverse health care systems. In the Beveridge ’public’ model, funding is based mainly on taxation and is characterised by a centrally organised National Health Service where medical/health services are provided by mainly public health providers (hospitals, community doctors, etc.). The Bismarck ’mixed’ model is funded mainly by a premium-financed social/mandatory insurance, and results in a mix of private and public providers. In the ‘private’ insurance model, funding of the system is based on premiums, paid into private insurance companies [24]. Mixed healthcare systems combine provisions of the Beveridge, Bismarck and Private insurance models. 

Welfare states and health care systems vary across the world. In the Scandinavian welfare state model, countries often have a Beveridge health care system and strong national institutions, where the coverage by health services is relatively universal and relatively well institutionalised. In the Bismarckian welfare state model with insurance-based health care systems, health services as a benefit is related to one’s employment and position in the labour market. Southern European welfare states have a mixed health care system either state-centred (e.g., Portugal, Greece) or bilateral (e.g., Spain, Italy), which can be decentralised towards regions and local communities. Anglo-Saxon welfare states differ in health systems, as some countries can be characterised by universal health services, while others can have mixed health care systems. In relation to the financing of welfare systems, most of the welfare or health care benefits are covered by taxation or insurance [18]. Table 1 presents the relations between welfare state and health care systems. Based on this applied taxonomy, the following countries were selected: Australia, Canada, Finland, France, Germany, Ireland, Italy, Japan, The Netherlands, Poland, United Kingdom and the United States of America. 

As part of the preparatory stage, the protocol was peer-reviewed and, subsequently, piloted. The piloting process involved the development and specification of one case study: the United Kingdom. The developed case study was externally and independently peer-reviewed for clarity, structure and alignment to key research questions. In addition, a reflective discussion within the research team of the employed protocol and its procedures occurred. Feedback received during this preparatory stage of the study was used to further develop, refine and finalise the research protocol. 

### 2.2. Data Collection

The data collection process included searching international- and national-level databases and information repositories and conducting targeted searches in the academic and grey literatures (e.g., ILO databases, reports from governmental bodies and national-level health and safety authorities, and academic data sources such as pubmed, sciencedirect, proquest, psyinfo and EBSCO). This included a review of regulation, policies, codes and guidelines relating to OHS. The review was not limited to specific legislation addressing OHS but also included general laws relating to occupational health and safety, labour law and social security, as well as information on the content, coverage and delivery of OHS at the national level. Collected information was indexed and summarised according to the following thematic areas: legal and policy context; content, organization and delivery of OHS; financing and coverage; minimum standards; the role of OH professionals; and employers’ responsibilities and national-level methods of support and incentivization. This review was, however, limited to the resources available in English and in the public domain. 

The data collection process yielded a case study collection of 12, and annotated index and summary of key case study characteristics. The annotated index categorised and summarised information extracted from the case studies by thematic area, and was used to support subsequent comparative content analysis (see next section). As a cross-validation method, national-level experts were recruited to critically review the completed case studies, assess the accuracy of the case study content and provide feedback on their presentation. 

### 2.3. Analysis

Following the completion of the case study collection and the accompanying annotated index, a comparative content analysis was conducted to identify key thematic codes and, in turn, identify and map convergent and divergent themes across the 12 case studies and within each specified thematic area. Thematic content analysis [25] was used to identify and explore key (core) themes across case studies within each respective thematic area. One researcher led on conducting the thematic comparative content analysis. Two further researchers reviewed extracted themes and codes for sense and accuracy. A process of reflexivity [26] supported by a series of group discussions within the research team was used to support this analysis process. These reflective group discussions included: reflecting on and reviewing data charts and research notes; comparing observed accounts, systems or identified characteristics of the reviewed case studies; discussing identified patterns and connections; and collectively exploring explanations for these internally within the data. 

## 3. Findings and Discussion

The analysis identified key policies, structures and approaches highlighting differences in financing, coverage and capacity of OHS systems across the 12 national contexts. The next sections present and discuss the findings of the comparative analysis across the three key themes identified: legal and policy context, organisation and financing of OHS, and coverage and staffing of OHS. 

### 3.1. Legal and Policy Context

The most elaborate infrastructure for occupational health practice, at the international level, is described in the ILO Occupational Safety and Health Convention, 1981 (C155) and the Occupational Health Services Convention, 1985 (C161) and its accompanying Recommendation (R171) [15]. Convention 155 outlines the basic principles of a national policy on workers’ health and safety and while it does not directly outline the requirement for OHS, it has the potential to indirectly lay the foundation for further health and work policies (including OH) to be developed. Of the 12 countries reviewed in this study, only 3 (Finland, Germany and Poland) had ratified Convention 161, while only 4 (Australia, Finland, Ireland and The Netherlands) had ratified Convention 155 (see Table 2). Despite this, we found that all countries included in this study had implemented many principles laid out in these conventions, and consequently have advanced infrastructures for the provision of OHS.

In spite of harmonisation efforts, wide variation is still observed between the national laws and practices stipulating OHS [18]. While some countries require provision of comprehensive services to all working people, some others require coverage only of those “in need” [13]. In most European countries, employers are obliged by law to organise OHS for their employees. This obligation is strictly enforced in countries such as Finland, France, Germany, Italy and Poland and still exists—albeit in a diluted form—in The Netherlands [27]. However, there is no corresponding legal requirement in the UK [28] or in Ireland (the Irish Health Services Executive does however provide a number of OHS). OHS in Japan is specified by law and sets out clear responsibilities for the employer, however no specific legal requirement on OHS exists in Australia, Canada, or the USA. 

Overall, there are two main types of legislation regulating OHS [15]. The first views the OHS as an integrated multidisciplinary service infrastructure and stipulates the objectives, activities, obligations and rights of the various partners, the conditions of operation, as well as the qualifications of its personnel. Within such national contexts, typically the delivery of OHS is stipulated through national-level legislation, for example in Finland, France, Germany, Italy, Japan, The Netherlands and Poland. The second type of legislation, as found in Australia, Canada, Republic of Ireland, UK and USA, is more fragmented and involves a number of laws that simply oblige employers to carry out certain activities. These may be stipulated quite specifically or merely in general, leaving issues of their organisation and conditions of operation open. 

In many countries, the laws governing the provision of OHS are incorporated into the national constitution and broad labour laws (e.g., in Canada, Finland, France, Italy, Japan, Poland), social security regulations (e.g., in Germany, France, The Netherlands, USA), human rights laws (e.g., in Canada, France), anti-discrimination laws (e.g., in Ireland, Italy, Japan, UK, USA), disability and rehabilitation laws (e.g., in Italy, Japan), as presented in Table 2. In many countries, instead of stipulating what might be regarded as OH programmes, the legislation stipulates the responsibility of employers to provide health risk assessments, health examinations of workers or other individual activities related to workers’ health and safety. The legislation usually delegates the authority to establish, implement and inspect OHS to such ministries or agencies as Labour, Health or Social Security [29]. 

Occupational safety and health (OSH) legislation in all 12 countries included provisions relating to OHS. Furthermore, other additional voluntary policies were identified in most countries on OHS and specifically related to psychosocial risk management and mental health promotion at work. These included laws, standards, codes of practice, sectoral agreements and guidelines. These findings are similar to those reported by previous research, which has highlighted that a number of policy approaches, both regulatory and voluntary, now exist in many countries around the world [30]. ‘Regulatory policies’ comprise regulations to promote occupational health and safety, tackle discrimination and promote equality (such as regional or national legislation, ILO conventions), while ‘non-binding/voluntary’ policies developed by recognised regional, national and international organisations include standards and specifications, guidance, recommendations and social partner agreements [31]. Some countries have enacted national laws, codes of practice and collective agreements to regulate psychosocial risks, setting employers’ responsibilities to deal with these issues [30,32], while certain labour inspectorates have designed procedures to enforce relevant regulations [33]. There is also some evidence which indicates that efforts have been made to share knowledge and develop competencies of key stakeholders in this area, such as inspectors [34] and occupational health services [18].

In Europe, one of the most concrete factors behind the development of OHS is the transposition and implementation of the Framework Directive 89/391/EEC on Safety and Health at Work, and in this context particularly its articles 5, 6, 7 and 14, which have implications for the tasks, methods and structures of OHS. Eight countries of the 12 countries examined in this study (at the time data was analysed) were member states of the European Union, including the UK. Articles 5 to 7 are primarily focused on, and situated within, the broader agenda of health and safety, as they have a concentrated focus on the surveillance of the workplace and work environment. Psychosocial risks and their management are among the employers’ responsibilities as stipulated in the Framework Directive as it obliges employers to address and manage all types of risk in a preventive manner and to establish health and safety procedures and systems to do so [35]. Despite this legal basis, the findings suggest that inclusion of issues relating to psychosocial risks, mental health and well-being at work in OHS provision is still limited due to the focus of OHS on traditional OSH issues and due to lack of awareness and expertise on management of psychosocial risks and promotion of mental health.

The findings also highlighted that at the national level, a number of European countries included in the study had implemented laws with specific clauses relating to psychosocial hazards at work, and made direct reference to mental health at work, work-related stress, psychosocial risks (e.g., Italy, The Netherlands, Finland, France, Germany), and all eight European countries had laws relating to the prevention of bullying and harassment at work. For instance, in Poland, harassment at work is covered by the Labour Code, while in the UK and Ireland, the Equality Acts place obligations on employers to prevent bullying/harassment in the workplace. While Japan and Australia have implemented national laws to prevent bullying and harassment at work, Canadian provinces have implemented their own laws, but in the US only four states (California, Utah, Tennessee, Puerto Rico) have relevant legislation in place and there is no Federal law which covers all workers [30,32,36,37]. 

Amongst all the countries examined in this study, the national legal context in relation to the management of psychosocial risk was most established in Finland and Italy. The objective of the Occupational Safety and Health Act in Finland is to improve the working environment and working conditions in order to ensure and maintain the work ability of employees as well as to prevent occupational accidents and diseases and eliminate other hazards from work and the working environment to the physical and mental health of employees. These policies also include specific reference to workload factors and exposure to violence at work. The objectives and principles of national level OSH activities include a focus on leadership as the corner stone of well-being at work, the importance of occupational health care and competence in relation to mental health and well-being at work [38]. Data over time (2005–2013) indicate a decrease in the number of all employees retiring on a disability pension due to mental health and behavioural problems and musculoskeletal disorders [39].

In Italy, Decree No. 81/2008 (modified by Decree No 106/2009), the primary OSH legislation, harmonised the OSH provisions across preceding regulations and sought to regulate the shared OSH competences between the Italian State and regions [40]. There are several notable novel aspects to this regulation which include unification of OSH legislation into a single text, inclusion of risk management programmes compliant with OSH management standards (e.g., ISO 45001) and significantly, inclusion of work-related stress in routine risk assessment according to the European Framework Agreement on work related stress of 2004 [41]. As regards the risk assessment of work-related stress, the Decree has assigned to the Permanent Consultative Commission for Occupational Health and Safety the task of developing specific guidelines to clarify the national legal framework (that was published in 2010 by the Ministry of Labour No. 30/2010) as methodological steps representing the minimum implementation level of the obligation. The main methodological approach used for the assessment and management of psychosocial risks in Italy is a methodology that complies with the Permanent Consultative Commission’s guidelines-developed by the Italian Workers’ Compensation Authority (INAIL, Rome, Italy) [42] which is also a key national stakeholder for OHS provision as discussed in the later sections of the paper. This methodology adapts and integrates the UK Management Standards for work-related stress model and provides a research-based systematic approach, and validated tools [43], allowing employers to manage work-related stress as integrated with all other risks for health and safety and involving in addition to occupational health and safety professionals, employees and their representatives [44]. This specific coverage of work-related stress in law and implementation of a specific methodology has more than doubled the number of enterprises reporting a procedure in place to deal with work-related stress, while more than tripled the number of enterprises reporting a procedure in place to deal with harassment or bullying, and with work-related violence [45,46].

Like in Italy, other countries have modified existing laws to include provisions relating to the management of psychosocial risks and promotion of mental health at work. In Germany, following intense political discourse, the German Occupational Safety and Health Act was amended in 2013, and now explicitly states that employers have to conduct a risk assessment including psychosocial risks and that measures implemented to follow up risk assessment have to consider both physical and mental health [47]. The French legal system recognises the employer’s duty to safeguard workers’ physical and mental health, under Article L. 4121-1 of the Labour Code and various legal provisions on health and safety at work. Decree No. 2001-1016 of 5 November 2001 imposed a duty on employers to evaluate risks at work within their organisation, covering both physical and psychosocial risks and the Uniform Occupational Risks Evaluation Questionnaire (DUER) became the first tool for investigation. The Labour Inspectorate has to verify this document in the course of inspections, and thus provides organisations and their OHS an early opportunity for evaluating risks before drawing up a prevention policy. In 2002, specific provisions on psychological harassment were introduced, thus providing a legal foundation for psychosocial risks at work most commonly cited in litigation [30]. 

Since 2012, Australia endeavoured to harmonise workplace health and safety laws across its States and Territories, developing a model set of laws which were to be adopted by each jurisdiction, as outlined in the harmonised Work Health and Safety Act (WHS Act) [36]. The WHS Act includes provisions to address psychosocial risks, and employers have a primary duty of care to their workers through the execution of various risk assessment and management procedures, and consultation with their employees, their representatives and other duty holders [48]. While in The Netherlands, laws on working conditions, which outline the general provisions for employers and employees on how to deal with health and safety at work, have evolved since the early 2000s, and since 2013 include specific provisions which require employers to pursue policies aimed at preventing or reducing work-related psychosocial risks including factors such as sexual harassment, aggression, violence, bullying, workload and pressure [49].

In Japan, social protection and health and safety laws have been amended several times since the 1970s and two areas of notable reform have been around the surrounding prevalence, and national-level concern, regarding *karoshi* (death brought on by overwork or job-related exhaustion) and *karo-jisatsu* (work-related suicide); however, despite these provisions, it is still difficult to obtain recognition of work-related mental health disorders and the employer’s liability, despite clear juridical rulings on this issue [30]. Two notable policy developments are the Silver Health Plan and the Total Health Promotion Plan, which have both focused on promoting workers’ physical and mental health. In particular, mental health promotion is a key priority in Japanese OHS [50]. In 2000, the Ministry issued guidelines on mental health promotion in the workplace. Employers are required to develop a mental health promotion plan with special reference to the system, its implementation, staffing and a privacy policy. They are also required to implement the plan through four routes: (1) self-care by employees; (2) care through line management, carried out by managers and supervisors; (3) care provided by the company’s healthcare staff; and (4) care provided by external healthcare staff [51].

Key non-binding national initiatives identified in the study include the Total Worker Health^®^ programme implemented in the US, the Management Standards for Work-related Stress in the UK, which have been adapted as the Work Positive approach in Ireland and also informed the development of the INAIL methodology in Italy, as discussed previously. National standards have also been introduced by standardisation bodies in Canada, Australia and the UK. To address issues relating to the management of psychosocial risks and promotion of mental health at work in the US, the Center for Disease Control and Prevention’s National Institute for Occupational Safety and Health (CDC/NIOSH) introduced the Total Worker Health^®^ (TWH) programme which outlines policies, programmes and practices that integrate protection from work-related safety and health hazards with promotion of injury and illness prevention efforts to advance worker well-being. TWH efforts, implemented by employers and their OH providers can help, protect the safety and health of workers and advance their well-being by fostering safer and healthier workplaces and by addressing work organisation, employment and supervisory practices and workplace culture [52].

The UK national legislative framework requires British employers to exercise a duty of care to their employees while at work, and in so doing ensure their health (including mental health), safety and welfare. In addition, under the Management of Health and Safety at Work Regulations, employers are required to carry out a suitable and sufficient assessment of significant health and safety risks, including the risk of stress-related ill health arising from work activities, and take measures to control that risk. The Health and Safety Executive’s (HSE, UK) Management Standards for Work-related Stress allows employers and their OHS providers to carry out risk assessment in relation to work-related stress, implement interventions and improvement of the work environment, and managing individual cases where needed [53,54]. The Work Positive^CI^ approach introduced in Ireland is a government and stakeholder supported psychosocial risk management process that helps organisations identify ways to improve employee well-being. It provides feedback on workplace stressors, employee psychological well-being and critical incident exposure in the workplace. It delivers structured guidance enabling organisations to develop an action plan to mitigate against these stressors. It is the first psychosocial risk management framework specific to critical incidents in Europe and incorporates an easy-to-use online risk assessment tool [55], that can be used by any organisation and OHS provider. 

In 2011, the British Standards Institution (BSI) developed the first guidance standard on psychosocial risk management in the workplace—PAS1010, to enable enterprises to address these risks as part of their occupational health and safety management systems [56]. In 2013, a national Canadian standard CAN/CSA-Z1003-13/BNQ 9700-803/2013 ‘Psychological health and safety in the workplace-Prevention, promotion and guidance to staged implementation’, was introduced. This voluntary standard specifies requirements for a documented and systematic approach to develop and sustain a psychologically healthy and safe workplace [57]. While in 2018, Safe Work Australia published ‘Work-related psychological health and safety—national guidance material’, which provides guidance to anyone (e.g., employers, OHS, etc.) who has a duty to prevent and manage harm to workers’ psychological health [58]. In 2018, a joint proposal by BSI (London, UK) and the Canadian Standards Association (Toronto, Canada), was put to the International Organization for Standardization (ISO, Geneva Switzerland) to develop an international guidance standard on psychological health and safety in the workplace, based on PAS 1010 and the Canadian standard. The proposal was approved and the new standard, ISO 45003 ‘Occupational health and safety management-Psychological health and safety at work: Guidelines for managing psychosocial risks’ is expected to be published in 2021. ISO 45003 will be the first global standard that will provide guidance on the management of psychosocial risk, as part of an occupational health and safety management system (e.g., ISO 45001) [59]. 

### 3.2. Organisation and Financing of Occupational Health Services

The legal and policy context, to a large extent, determines the predominant OHS model and their financing. To meet the occupational health needs of enterprises, which vary widely with respect to type of industry, size, activity, structure, a number of different models of OHS have been developed [13]. While the most advanced OHS are in concordance with the ILO instruments, other types of infrastructures may be used. The OHS may be a single integrated entity, or a composite of different occupational health and safety units unified by a common concern for workers’ health and well-being [15]. Organisational and financing models for OHS vary between and within countries, according to their: national traditions, legal and policy context, the organisation of occupational health and safety, the health system, social security and industrial and economic activity [29]. In general, the way OHS are organised and financed reflects the interests of the government and social partners in relation to the health and well-being of the working age population [13]. 

Table 3 presents a summary of the way in which OHS are organised, delivered and financed, in each of the 12 countries examined in this study, with results indicating that many countries use ‘mixed OHS models’. In all 12 reviewed countries, the employer bears (either full or partial) financial responsibility for the provision of OHS, including countries where employers have a legal duty to provide such provision and services. Employers may directly fund such systems by either in-house, group (e.g., in Japan, France) or externally contracted OHS (e.g., The Netherlands, UK, USA) or they may pay levies or contributions which go to support group services models (e.g., Germany, Poland). In many of the reviewed case studies, the state also plays a key role in financing OHS through social security agencies (e.g., Germany), specialised agencies in OH (e.g., Poland) or the healthcare sector, for instance in Italy and Ireland. The bottom line commonly seems to be that the funding of OHS is derived from the profits and production of industry and services. This applies equally in the public and private sectors. In these countries, citizens/taxpayers are apparently not major stakeholders where the funding of OHS is concerned [27]. 

The in-house (or in-company) model refers to the range of integrated OHS available within enterprises (typically large) in both the private and public sectors, while the group or inter-enterprise or industry model refers to sharing of OHS by groups of small and medium-sized enterprises (SMEs) and has been widely used in industrialised countries. This enables enterprises that are individually too small to have their own services, to enjoy the advantages of a well-staffed, well-equipped comprehensive service. In some instances, OHS make arrangements with local hospitals to provide certain specialised services that are comparable to in-plant or group services [15]. British employers typically provide OHS through three key avenues: in-house; direct appointment; or through competitive tender [60] and studies suggest that smaller employers are much less likely to have in-house OH provisions or services compared to larger employers [61]. 

In some countries, employers of a certain size are required by law to provide an in-house service [29], as found in Japan, France and Poland. In Japan, larger enterprises (employing more than 1000 employees) have to establish their own in-plant OHS while medium-sized and small enterprises are required to join group services [51], while in France, there are two broad types of OHS: OH ‘group service enterprises’ (or inter-company services, generally for smaller sized companies) and ‘autonomous’ (in-house) OHS run by an individual company. The choice of delivery method is largely made according to the number of employees to be covered. All OHS are regulated by law and overseen by committees representing employers and employees [62]. In 2011, legislative amendments extended the role of OHS to preserving physical and mental health, by advising employers, workers and their representatives, mitigating occupational risks, improving working conditions, preventing excessively harsh working conditions and exclusion from work, and general health monitoring [63]. In Poland, employers are also obliged to put in place and fund a Work Safety and Hygiene Service that operates within companies and is, in general, responsible for advising employers on all aspects of work safety, health and hygiene. Depending on the size of the enterprise, the employer can create the service (100+ employees), enlist a competent person to undertake respective duties (less than 100 employees) or take such responsibility themselves. Alternatively, the employer may contract an external expert (which is often the case) [64]. 

The goal of health care in Finland is to ensure the psychological and physical functional health through preventative-focused, comprehensive health care services. The Finnish health care services are spilt into primary health care and specialised medical and hospital care. Public Health Services include six elements, with OH services being one such aspect [65]. There are four different models of OHS used in Finland. The employer can acquire OHS from: municipal health centres; private medical centres; an OHS unit integrated into the enterprise; or enterprises can jointly organise their OHS. However, there has been an increase in the number of private OHS providers in Finland since the early 2000s [66]. OHS are provided by law, and the occupational health care system supports workplaces in their risk assessments and in improving workplace well-being. The provision of the services is supervised, and their availability is promoted [38]. Similarly, for many decades, the primary delivery point of OHS in the US were on-site facilities at companies that wanted to provide such services to their employees. However, in recent decades there has been a change from the traditional in-house health unit to the utilisation of community resources (OHS in hospitals, medical centres or private clinics), or the intermittent assistance of private consultants [67], as also seen in The Netherlands, where even though the provision of OHS is specified by law, it is predominantly market-driven. Mandated OH support can be delivered by an externally contracted OHS or by hiring in only specific expertise (customised support) [68]. 

In the social security model, OHS are provided by special units organised and operated by the social security system. While this model can be similar in structure and operation to the group model, its specific feature is that it is operated by the organisation responsible for workers’ compensation for occupational injuries and diseases. While curative and rehabilitative services are provided, the emphasis on controlling social security costs often leads to priority being given to preventive services [15]. In this study, examples of the social security OH model were found in Germany and Italy. In Germany, OHS are provided by: in-house services in larger enterprises; contracted external services from the Association of German Accident Insurance Funds; other local, regional or nationwide services in private ownership; or by independent OSH professionals. All operate in accordance with the minimum time specifications by the insurance fund in charge of the firm. Social accident insurance institutions (e.g., DGUV) also offer occupational safety and medical services in some sectors [69]. The institutional responsibility for providing workplace health promotion rests entirely with the statutory health insurance funds and not with the occupational health and safety authorities or other public agencies, and in 67% of documented cases, such measures are (at least partly) aimed at the improvement of working conditions [70]. While in Italy, the law gives the Italian Workers’ Compensation Authority (INAIL) the responsibility for the reintegration of persons with disabilities caused at work, through the implementation of projects that provide retraining and upskilling; adaptations of work-stations and workspaces, or support the identification of a new position [71]. To address work-related stress, and in accordance with current regulatory frameworks, INAIL has also developed a web platform for all public and private Italian organisations, that provides tools for assessing work-related stress risks and guidance on designing and implementing interventions [44].

The healthcare system in many national contexts is important in providing ill, disabled or injured workers with access to OHS. In some countries this system is offered through specialised OH agencies (e.g., Poland), integrated into primary health care (e.g., Italy, Ireland), or some basic OHS provided are publicly funded and free at the point of access (e.g., the UK, Canada and Australia), and others are privatised (the USA). Therefore, the government, insurers or the workers themselves are key financial contributors. For instance, in Poland, the Ministry of Health is responsible for creating Occupational Medicine Services (OMS), which provide a range of OHS and are responsible for ensuring the safety, hygiene and healthiness of employees’ work and their work environments. OMS operate, independently from any employer established services, in a two-level structure with primary OH centres and regional centres. Primary OH centres are typically health care institutions (whether public or private) and provide preventive health care at a workplace, while regional centres are public healthcare institutions established by a regional government, and are generally responsible for inspecting and monitoring primary centres, and for providing them with expert advice and supporting them in their activities [72].

In Italy, OHS are integrated into primary health care where each regional health authority provides OHS, with the mandate of enforcing the application of the Italian OSH law and sanctioning violations within their region. These units offer a combination of medical surveillance, occupational and environmental hygiene, risk assessment and regulatory activity [41]. While in Ireland, the Irish Health Services Executive (Dublin, Ireland) provides a number of OHS which are embedded within Community Health Organisations and Hospital Groups. Pathways to access such nationalised OHS include self or management referral. Each OHS is staffed by an occupational physician, OH nurses and an OH administrator. Beyond the services offered through the healthcare system, an estimated 45% of Irish employers have OHS in place, whether that is internal or external to the company. The provision of such OHS are, however, more common in large organisations [73]. 

Integrating OHS into a primary health care unit is the model most recommended by the WHO as a means of providing services to all enterprises, particularly SMEs, those in the informal sector and the self-employed. Since general physicians and nurses usually lack specialization and experience in occupational health, and particularly mental health at work, the success of this model critically depends on how much training in occupational health and occupational medicine can be arranged for the health professionals [15]. In countries such as Australia, Canada and the UK, when a worker is injured or ill they can use a treating doctor of their choice, typically their General Practitioner (GP). Communication to the employer and insurance company is by a medical certificate (e.g., a sick note or a fit note), which is issued by the treating doctor. The content of the certificate encourages the doctor to outline what duties the worker should be restricted from performing. A limitation of such a system is that treating doctors do not have a global view of working conditions, only those discussed or highlighted by the injured or ill worker. Consequently, such limited knowledge may have an impact on the effectiveness or indeed suitability of the injury management plan [74]. OHS in the UK are not directly or purposively integrated within current National Health Service provisions, albeit employees may receive some OH-related medical care through primary or secondary care. However, the growing shortage of OH physicians and nurses has added to recent calls for the specialty to be integrated fully within the NHS [75].

### 3.3. Coverage and Staffing of Occupational Health Services

OHS are unevenly distributed in the world [12,22]. In Europe, about half of the working population does not have access to competent OHS, and the variation among countries is very wide, with coverage figures ranging between 5% and 90% of the workforce [76]. The countries that strictly enforce the provision of OHS can demonstrate high rates of coverage of the workforce; however, in most countries, reliable assessments are difficult due to statistical uncertainties [27]. Lower coverage figures are found on other continents. Only a few countries (Australia, Canada, Japan, USA) show coverage figures comparable to those in Western Europe. Even in countries where coverage rates are high, there are gaps, with SMEs, certain mobile workers, construction, agriculture and the self-employed being underserved [50,77]. Table 4 presents the coverage of OHS, in each of the 12 countries, with results indicating that coverage varies from as low as 30% in Ireland and USA, high coverage in Finland, France, Italy, Japan, The Netherlands and near full coverage in Germany and Poland which provide OHS to most of their working population.

Traditionally, OHS have been staffed by occupational physicians (OPs) only, or a physician and a nurse who, perhaps with the addition of an industrial hygienist, may be designated as the “core” staff [15]. Most recent provisions, however, require that whenever possible the OH staff should be multidisciplinary in composition. The staff may be enlarged to a full multidisciplinary team depending on the model of the service, the nature of the industry and the types of work involved, the availability of various specialists or of programmes for training them and the extent of the available financial resources [13]. They may include safety engineers, organisational/mental health specialists (e.g., psychologists, counsellors), work psychologists, ergonomists, physiotherapists, toxicologists, epidemiologists and health educators. Most of these are rarely included in the full-time staff of the OHS and are often involved on a part-time or an “as needed” basis [15]. 

In France, OPs according to article L. 4624-1 of the French Labour Code, are required to propose individual measures to the employer to protect the mental health of an employee, depending on his/her condition [30]. In addition to this, a move towards multidisciplinary service providers has been a key focus of OHS reforms in France since the early 2000s, which gave a more multidisciplinary steer to its national-level OH system. The aim was to deliver primary risk prevention by supporting OPs with other medical and allied (health) professionals (e.g., OH nurses, ergonomists, psychologists, toxicologists, etc.). However, this became a legal requirement only in 2012, and improved collaboration within OHS, and exploitation of a wider range of professional competencies and skills are still needed [83]. Similarly, in Germany, OHS are usually multidisciplinary offering specialist interventions from an increasing number of professions. All major OHS organisations employ at least an OP, a safety engineer and a psychologist, however, the range of the services on offer increases with the size of the enterprise [69]. There are also some challenges in bridging health at work and general health issues, a need to improve cooperation between OPs and general health care providers, considerable gaps in availability of support by OPs and OH experts, especially in some sectors and SMEs, and also less availability of psychosocial risk expertise [70].

OPs and OH nurses are key actors in the delivery of OH provisions across jurisdictions in Canada. However, it is primarily the employees’ GPs who are at the frontline in the delivery and co-ordination of medical and rehabilitative care. Other professionals (psychologists, ergonomists and occupational hygienists) are also key professionals in the field, but co-ordination of such multidisciplinary teams is varied and fragmented across jurisdictions. In general, the private practice of occupational medicine has become a major growth area in both the US and Canada [84]. In Australia, injured or ill workers may receive support from return-to-work practitioners. These practitioners coordinate with the employer and the treating doctor but may also provide some level of assessment (e.g., ergonomic) of the workplace. Such practitioners may be from a variety of disciplines such as psychology, physiotherapy, occupational therapy [74]. In the UK, OHS are typically staffed and supported by OH physicians and nurses, although a wider spectrum of professionals may be required to support additional more specialised services or provide tailored advice (e.g., psychologists, ergonomists, occupational hygiene specialists, etc.) [85].

The role of various professions in managing psychosocial risk was only found to be clearly defined in Italy, where the national approach comprises the establishment of a multidisciplinary Steering Group and the use of two tools [42]. The first is a checklist to carry out a preliminary assessment, of objective indicators such as injuries, sick leave absence, other absence from work, turnover, formal records of employees’ complaints to the company or to the company’s occupational physician [86]. In addition, the second part of the checklist is used to identify psychosocial risks on the basis of group discussions with workers that have specific work-related risk factors and organisational aspects in common. This information is collected from organisational records by the Steering Group that includes the employer or his/her representative, a health and safety manager working for the organisation and his/her staff, the occupational physician(s) and the employee representatives. The Steering group may include other professionals when present in the organization, such as psychologists [87]. In addition, Japan is a unique national example where obligations are set out for employers and several concrete guidelines have been issued by the Ministry of Health, Labour and Welfare, e.g., guidelines to prevent *karoshi* and to promote mental health in the workplace [51,88]. Personnel involved in health promotion programmes include health educators, mental health advisers and healthcare trainers [51].

The professional identity of OH specialists needs to be supported on an equitable basis among the various disciplines [15]. In most countries, there are intentions to develop the competences of OH professionals and to increase the level of multi-disciplinarity. Presently, however, the medical professions are predominant in most countries, and the focus has generally been on the provision of curative services rather than on preventive action [27]. This study also found that in many countries, despite the importance given to multidisciplinary OHS, the focus still remains on OPs. For instance, even though the use of multidisciplinary OHS is encouraged in Japan, nothing is stipulated in the Industrial Safety and Health Act concerning ergonomists, industrial hygienists or psychologists [51], and while in Poland OHS comprise different vocations, such as OP, OH nurse, industrial hygienist, psychologist, psychotherapist, ergonomist, public health specialist, general physician, etc., there is no definition of an OH professional [89]. 

Similarly, in The Netherlands, by law there are four key professionals that are central to the delivery of OHS, OPs, safety officers, occupational hygienists and work and organisational professionals. Work and organisational professionals should have expertise in optimisation of work content or organisation of work, development and implementation of OSH systems and the development, improvement and implementation of policy on absenteeism. Each of these professions is supported by their own professional association. Other specialists not specifically mentioned in the law but who may also be actively involved in the multidisciplinary delivery of OHS are OH nurses, ergonomists and psychologists [68]. While in Finland, the core team of many OHS includes an OP and OH nurse, and in many units, a physiotherapist and psychologist also belong to this team. However, the composition of multidisciplinary services across OH units tends to vary greatly, and among these additional specialists, the training for OH nurses, physiotherapists, psychologists and other experts has not received the same level of national attention as that of OPs [90].

## 4. Conclusions: Key Development Needs of OHS to Ensure Management of Psychosocial Risks and Promotion of Mental Health at Work

While the findings of this study highlight the similarities and variations in the context, systems, structure, coverage and capacity of OHS, that have also been reported by other comparative studies of OHS systems [12,27], they have also enabled the identification of key development needs of OHS to ensure psychosocial risk management and mental health promotion are prioritised effectively in a preventive manner. The findings clearly demonstrate that the development and enhancement of OHS, and inclusion of clear objectives relating to the management of psychosocial risks and promotion of mental health and well-being at work at the national level, are essential for managing the needs of health and work ability of the working population in this changing world of work [1,12]. 

The findings from this study highlight that while most countries have laws governing the provision of OHS, the structure of the legislation, its content and the workers covered by it vary widely [12,22]. Similarly, in most countries included in the study, mental health and psychosocial risks in the workplace have been recognised as priorities in occupational health and safety, and a number of hard and soft law policies of relevance to them have been developed over the years that have promoted awareness and action among social partners, organisations and indeed individual workers [31]. However, there are still significant gaps in implementation, capacity and coverage of these provisions as also highlighted by Rantanen and colleagues [22], and as well as lack of integration of OHS provisions with those relating to the management of psychosocial risks and promotion of mental health. 

### 4.1. Implementation Gap

While infrastructures for the provision of OHS are relatively developed in all countries examined in this study, the implementation of strategies remains insufficient in many. In addition to OHS provision in OSH legislation, specific laws exist in some countries (e.g., France, Finland, Germany, Poland), which regulate the activities of OH services, determine the nature of provision (i.e., medically oriented vs. multidisciplinary, etc.) and stipulate roles and responsibilities of various stakeholders. However, the lack of a legal basis in many countries, creates divergence in practice with several employers providing their workers no access or only limited access to OHS. Even in countries where there is an advanced legislative basis for OHS and OSH, there can be a lack of coordination between different agencies/stakeholders involved in provision of OHS, and this particularly impacts new and emerging forms of occupational risks such as psychosocial risks.

It can also be observed that the introduction of different types of policies, such as legislation, guidance and national standards have helped in clarifying national legal frameworks, and employer and employee responsibilities, and spurred organisational action [3,34,35]. However, there is limited specific legislation and guidance on psychosocial risk management and mental health promotion for OHS with a clear focus on the implementation of a preventive framework of action. A more coordinated action plan would therefore be beneficial for clarifying requirements for OHS drawing upon good practice efforts. As specified in ILO Convention 161, OH services should focus on preventive functions, advising the employer, the workers and their representatives in the undertaking on the requirements for establishing and maintaining a safe and healthy working environment which will facilitate optimal physical and mental health in relation to work, and the adaptation of work to the capabilities of workers in the light of their state of physical and mental health [91]. While some countries have made progress towards these goals, there is still a long way to go for most.

### 4.2. Coverage Gap

While the estimated coverage of OHS was found to be as low as 30% in some countries examined in this study, the majority reported high coverage, with some also reporting their entire employed population having access to OH services. To some extent, the discussion on coverage reflects whether OH is regarded as a public health or a market commodity. If it is a matter of public health, full coverage of the labour market would be sought. However, countries which lean towards market mechanisms to provide coverage, might put less weight on the public health aspects and might give low priority to achieving high degrees of access to OHS organisations [92]. However, the current findings indicate that this might not be the case, as even in countries where OHS provision is market-driven, such as The Netherlands, there is evidence of high coverage. 

However, it is important to highlight the potential coverage gap in relation to prevention rather than treatment and rehabilitation, and the need for multidisciplinary services when implementing measures to manage psychosocial risks and promote mental health and well-being at work. For instance, a study of Finnish risk assessment practice that included OHS [93], found that only 46% of the respondents (representatives of OHS n = 469) indicated that risks of accidents, physical and mental stress factors, as well as physical and chemical risk factors were considered well in their assessments. Other studies have also indicated similar findings. For instance, a study examined the extent to which employers include work-related psychosocial risks when carrying out workplace risk assessments as required by law. Using data from 6500 German companies, the authors found that the prevalence of psychosocial risk assessments was only 21%, with large deficiencies identified in small companies [94]. Therefore, while in earlier discussions of OHS systems, coverage was commonly seen as an important objective in itself, the focus over time has shifted more towards questions of the capacity and quality of the service, its producers and performance, and its compliance with stakeholder expectations [95,96]. 

### 4.3. Capacity Gap 

While the content and multidisciplinary nature of OHS in the countries examined broadly corresponds to international guidance, the coverage, comprehensiveness and content of services remain largely incomplete due to a lack of infrastructure and shortage of multi-professional human resources [22] creating a capacity gap. Several studies have identified a clear need for additional capacity building in terms of education, training, tools and expertise. This does not only concern the organizational level but also policy making since studies have found a strong association between low awareness of policy makers and prioritization of psychosocial risks and mental health at work at various levels (e.g., [31,97]). This can negatively affect social dialogue at the national level and hinder both agreement among the social partners as well as the implementation of appropriate actions to tackle psychosocial risks and work-related stress [97,98].

Interestingly, in research conducted in countries where there is already a higher level of awareness and engagement of businesses in this area, it has been found that additional support is needed [99], especially to ensure continuous improvement of the psychosocial work environment. In addition, it appears that employers report greater satisfaction with support provided by independent experts in some countries than with that provided by national institutions [99]. Hence, there is a need for the provision of further competency development of OHS in relation to psychosocial risk management and mental health promotion whose role might be crucial in engaging organizations, and especially SMEs. Overall, better coordinated efforts are required among stakeholders to achieve desired results. Provision of easily accessible training to all stakeholders is important for achieving these aims [3].

## Figures and Tables

**Table 1:** Relations among welfare state and health care systems.

	Beveridge Health Care System	Bismarck Health Care System	Mixed Health Care System
Scandinavian welfare state model	e.g., Denmark, Finland, Sweden, Norway		
Bismarckian welfare state model		e.g., Austria, Germany, The Netherlands, France, Belgium, Luxembourg, Poland, Japan	
Anglo-Saxon welfare state model	e.g., UK, New Zealand		e.g., Australia, Ireland, Canada, USA
Southern-European welfare state model			e.g., Spain, Portugal, Italy, Greece

Source: Adapted from Hämäläinen [18].

**Table 2:** Legal and policy context relating to occupational health services (OHS), psychosocial risks and mental health at work.

Country (ILO Conventions on OHS Ratified)	National Regulation/Policies
**Australia** C155 in 2004	Model Work Health and Safety Act; Model Work Health and Safety Regulations; National Employment Standards; Fair Work Act; Workers’ Compensation Law; Model Codes of Practice; National Compliance and Enforcement Policy; Work-related psychological health and safety: National Guidance Material.
**Canada**	Canada Labour Code; Canada Occupational Health and Safety Regulations; Occupational Health and Safety Act; Occupational Health and Safety Code; Employment Equity Act; Canadian Human Rights Act; Government Employees’ Compensation Act; National Standard of Canada for Psychological Health and Safety in the Workplace.
**Finland** C155 in 1985 C161 in 1987	Finnish Constitution; Occupational Safety and Health Act; Act on Occupational Health Services; Act on Occupational Safety and Health Enforcement and Cooperation on Occupational Safety and Health at Workplaces; Government Decree on the principles of good occupational health care practice, the content of occupational health care and the qualifications of professionals and experts; Government Decree on medical examinations in work that presents a special risk of illness; Occupational Accidents Insurance Act; Act on Occupational Diseases; Employment Contracts Act; Working Hours Act.
**France**	Labour Code; Public Code; Society Security Code; Law 2011-867 on the organisation of occupational medicine; Decree 2012-135 on the organisation of occupational medicine; Decree 2012-137 on the organisation and operation of occupational health services; Law 2014-40 on sustainability and justice of the pension system; Decree 2016-1908 on modernisation of occupational health services.
**Germany** C161 in 1994	Occupational Health and Safety Act; Act on occupational physicians, safety engineers and other occupational health and safety specialists; Works Constitution Act; Ordinance on Workplaces; Ordinance on Occupational Diseases. National regulations and acts are supplemented by prevention regulations developed and implemented by social accident insurance institutions.
**Ireland** C155 in 1995	Safety Health and Welfare at Work Act; Safety, Health and Welfare at Work Regulations; Industrial Relations Act; Unfair Dismissal Acts; Employment Equality Acts; 19 published codes of practice; Standards for Occupational Health Services.
**Italy**	Italian Constitution; Law 833/78 Establishment of the National Health Service (also known as the Health Reform); Legislative Decree no. 151/2015; Decree No. 81/2008 on Health and Safety at Work and subsequent modifications and integrations; Legislative Decree No. 19/2014 “Implementation of Directive 2010/32/EU to the framework agreement, concluded by HOSPEEM and EPSU, on the prevention of needlestick and sharps injuries in the hospital and health sector; Decree 38/2000 on list of recognised occupational diseases and occupational disease insurance; Decree 1124/1965 on health care assistance; Law 190 of 2014 on INAIL’s responsibility for the reintegration of persons with disabilities caused at work; Legislative Decree no. 215/2003 and Legislative Decree no. 216/2003 on rights to equal treatment; Law n. 68/1999 (& its DPR 10.10.2000 n. 333) on the right to work of persons with disabilities.
**Japan**	Constitution; Labour Standards Law; Trade Union Law; Labour Relations Adjustment Law; Industrial Safety and Health Law; Ordinance on Industrial Safety and Health; Workers’ Accident Compensation Insurance Law; Ordinance on the Payment of Special Supplements of Workers’ Accident Compensation Insurance; Equal Employment Opportunity Act; Law on the Elimination of Discrimination against Persons with Disabilities; Act for Promotion of Employment of Persons with Disabilities.
**The Netherlands** C155 in 1991	Working Conditions Act; Working Conditions Decree; Working Conditions Regulations; Procedural Regulations in the Working Conditions Decree; 1966 (disability pension for employed persons); 1998 (disability pension for self-employed persons); 1998 (disability assistance for young persons); 2006 (disability pension for employed persons); 1964 (medical benefits); 1966 (sickness and maternity benefits); 1968 (exceptional medical expenses); 2005 (health insurance); Work and Income (Employment Capacity) Act; Gatekeeper law; OSH catalogues.
**Poland** C161 in 2004	Constitution of the Republic of Poland; Labour Code; Acts on National Labour Inspectorate and on Social Labour Inspection; Act No. 593 on Occupational Health Services; Regulation on general provisions for safety and health at work; Regulation on carrying out medical check-ups for employees, scope of preventive health care for employees and medical statements issued for purposes specified in the Labour Code; Ordinance on work safety and hygiene service 1997; Ordinance on work safety and hygiene training; Ordinance on occupational diseases; Regulation on the procedure for drawing up documents on occupational diseases and their aftermaths; Occupational Medical Service Act, 1997.
**UK**	Health and Safety at Work Act 1974; Management of Health and Safety at Work Regulations 1999; Employers’ Liability (Compulsory Insurance) Act 1969; Workplace (Health, Safety and Welfare) Regulations 1992; Health and Safety (Consultation with Employees) Regulations 1996; Reporting of Injuries, Diseases and Dangerous Occurrences Regulations (RIDDOR) 2013; Equality Act 2010; Approved Codes of Practice (ACOPs); Management Standards for Work-related Stress; Guidance standard on psychosocial risk management in the workplace–PAS1010.
**USA**	Occupational Safety and Health Act; Environmental Protection Act; Occupational Safety and Health Act Regulations; Fair Labour Standards Act; Workers’ compensation regulations; Family and Medical Leave Act; Americans with Disabilities Act; Civil Rights Act; Social Security Act; Rehabilitation Act; Age Discrimination in Employment Act; Affordable Care Act; Federal Insurance Contributions Act; Self-Employment Contributions Act; Ticket to Work and Work Incentives Improvement Act., Total Worker Health^®^.

**Table 3:** Models and financing of occupational health services.

Country	National OHS Context	Predominant OHS Models	Key Funders
**Australia**	Voluntary, outsourced and market-driven. Operating within an Anglo-Saxon welfare model, mixed healthcare system.	In-house model, private OH provider, social security model, hospitals and clinics.	Employers, state and Social Security Programme.
**Canada**	Voluntary, outsourced and market-driven. Operating within an Anglo-Saxon welfare model and mixed healthcare system.	In-house model, private OH provider, social security model, hospitals and clinics.	Employers and the state.
**Finland**	Specified by law and mixed (state provision, and some market-driven provision). Operating within a Scandinavian welfare state model and Beveridge healthcare system.	Primary health care units, private OH provider, in-house model, group model.	Employers, regional authorities, Social Security Programme.
**France**	Specified by law and mixed (state provision, and some market-driven provision). Operating within a Bismarck welfare and healthcare system.	In-house model, group model.	Employers, regional authorities (funded by employer contributions).
**Germany**	Specified by law and market driven. Operating within a Bismarck welfare and healthcare system.	Social security model, in-house model, group model.	Employers.
**Ireland**	Voluntary, outsourced and market-driven. Operating within an Anglo-Saxon welfare state model and mixed healthcare system which provides several OH services.	Primary health care units, in-house model, community-based health centres, social security.	Employers and the state.
**Italy**	Specified by law and integrated with primary healthcare. Operating within a Southern-European welfare state model and mixed healthcare system.	Primary health care units, and specialist insurance agency.	Employer and regional authorities.
**Japan**	Specified by law and mixed (state provision, and some market-driven provision). Operating within a Bismarck welfare and healthcare system.	In-house model, private OH provider, community-based health centres.	Employers and the state.
**The Netherlands**	Specified by law and market-driven. Operating within a Bismarck welfare and healthcare system.	In-house model and private OH provider.	Employers.
**Poland**	Specified by law and mixed (state provision, and some market-driven provision). Operating within a Bismarck welfare and healthcare system.	In-house model and primary health care units.	Employers and the state.
**UK**	Voluntary, outsourced and market-driven. Operating within an Anglo-Saxon welfare state model and Beveridge healthcare system.	In-house model, private OH provider, within-hospital services for hospital staff (with hospital services provided to employers).	Employers, state, and Social Security Programme.
**USA**	Voluntary, outsourced and market-driven. Operating within an Anglo-Saxon welfare model and mixed (predominantly private insurance model based) healthcare system.	In-house, private OH provider, in-house model, social security.	Employers, state and Social Security Programme.

**Table 4:** Coverage and staffing of occupational health services.

Country	Estimated Coverage	OHS Staffing
**Australia**	50% of workers have access to some form of OHS [78].	No nationalised system of OH for members of the public. Injured/ill worker can use their GP who issues certificate of capacity, provides medical treatment and medication, recommends periods of time off work, advises on compensable medical and care treatments necessary for recovery and makes decisions that impact on the liabilities of compensation agencies. Return-to-work practitioners, rehabilitation providers (e.g., psychologists, physiotherapists, occupational therapists) often employed to facilitate or expedite process; co-ordinate with the employer and treating doctor; may also provide some level of assessment of the workplace. If a worker is not rehabilitated, an approved medical specialist with training and certification in disability assessment, calculates permanent disability.
**Canada**	48% of Canadians have access to workplace OHS. 66% have private health insurance, which supports provision of some basic OHS [79].	GP or other healthcare practitioner (certified nurses, physiotherapists, occupational therapists and chiropractors) provide medical certification for illness/injury, oversee and co-ordinate treatment. Disability Management Advisors: primary source of support for injured/ill workers, oversee case management and facilitate remain-at-work or return-to-work process. Compensation advisors: provide information to workers on benefits and the options to maintain income if injured/ill. Case managers with the provincial workers compensation board: determine whether the worker’s claim is accepted and entitlement to benefits, and facilitate remain-at-work or return-to-work process.
**Finland**	The coverage of OHS is about 90% of all Finnish employees [22].	Finnish public health services include OHS which are preventative. Employers can acquire OHS from municipal health centres or private medical centres; services may be integrated into the enterprise, or enterprises can jointly organise their OHS. OH physicians provide primary care and are the employee’s GP. They take action to improve health and safety, as well as employment relations, welfare, productivity and working life (namely, working environment, management and organisation). OHS include OH physician and OH nurse, and in many cases also a physiotherapist and psychologist. Other experts used when needed: ergonomists, occupational hygienists, construction engineers, agriculture advisors, opticians, dieticians, speech therapists and physical fitness trainers.
**France**	More than 90% of the workforce have access to OHS. OHS compulsory for all private and public sector organisations, but this excludes the self-employed [62].	Two types of OHS: OH ‘group service enterprises’ (or inter-company services, generally for SMEs, non-profit) and ‘autonomous’ (in-house) OHS run by an individual company. Occupational Physicians (OPs) have central role in both types of OH services, are independent and have protected status within the system. Reforms since 2000 gave a more multidisciplinary steer to the national-level OH system, with the aim to deliver primary risk prevention by supporting OPs with other medical and allied health professionals (e.g., OH nurses, ergonomists, psychologists, toxicologists, etc.).
**Germany**	Near comprehensive coverage [70].	All workers are insured and have access to OHS, largely provided by accident insurance institutions, which cover medical and occupational rehabilitation and provide compensation to those suffering from occupational diseases or injuries. OHS are multidisciplinary employing at least an OP, a safety engineer and a psychologist.
**Ireland**	30–40% of Irish workers have access to OHS [80].	Health Services Executive provides OHS embedded within Community Health Organisations and Hospital Groups. Two pathways to availing OHS within this system: through a self-referral or via a management referral route. Each OHS is staffed by an OH Physician, OH Nurses and an OH administrator.
**Italy**	Over 75% of the Italian workforce is covered by OHS [22]. This is, in part, due to the integration of OHS with primary health care through the regional health authorities.	According to Art.2 of the L.D. 81/08, employers are obligated by law to appoint competent OPs to carry out employee health surveillance. OPs must transmit collective data to INAIL and communicate such data to other organizational prevention officers. Most OPs are self-employed consultants, who may or may not be associated with agencies providing OHS and only a small proportion are permanent in-house employees. Most OH departments in University hospitals also provide OH consultation and diagnostic services. OPs collaborate with the employer and with the prevention and protection service to conduct yearly risk assessment, with the purpose of planning and conducting, where necessary, health surveillance on workers exposed to specific risks for health and safety and health promotion programmes mainly implemented in large organizations. They also assess fitness for a specific job. However, sickness absence with a length lower than 60 days is directly managed by the GP, and his/her certification is subject to verification by the specific public health office.
**Japan**	85% of Japanese workers have access to OHS [22].	Workplaces employing 50 or more workers are obliged to appoint an OH physician. Large-scale workplaces often provide full-size OH units that OPs are directing and may include general nurses, OH nurses and medical technologists. In SMEs, part-time OPs are recruited from among private GPs, hospital- or university-affiliated physicians and independent OH consultancies. Regional Occupational Health Centres and Occupational Health Promotion Centres, established by the government, provide health guidance for employers or employees and information on OHS, free of charge.
**The Netherlands**	80% of Dutch workers have access to OHS [80].	Employers can provide in-house services or contract external OHS, or hire only specific expertise to address specific OH issues identified by the organisation. By law there are four key professionals that are central to the delivery of OHS: OPs, safety officers, occupational hygienists and work and organisational professionals.
**Poland**	All active workers have access to OHS, resulting in near comprehensive coverage [81].	OHS are provided by a two-level structure with primary and regional OH centres. Primary OH centres can have various organisational structures. Physicians with adequate qualifications can either accept employment in a healthcare institution which provides OHS or run their own practice. OHS comprise: OH physician, OH nurse, OH hygienist, psychologist, psychotherapist, ergonomist, public health specialist, GP, etc.
**UK**	51% of British employees have access to OHS [82].	GPs have central role in diagnosing work-related ill health or injury. Employers have 3 main options of OHS: in-house; direct appointment; and competitive tender. OHS provided through a mix of private and NHS-led services. Occupational Safety and Health Consultants Register (OSHCR) established in 2010 to assist businesses in finding advice.
**USA**	35% of the US workforce is covered by OHS [22].	Employers particularly in the manufacturing sector use in-house health units as well as community resources (OHS in hospitals or medical centres or private clinics). Most OHS provision is through private consultants or external OHS providers.

## Data Availability

Not applicable.
